# Induction of Granulysin and Perforin Cytolytic Mediator Expression in 10-Week-Old Infants Vaccinated with BCG at Birth

**DOI:** 10.1155/2011/438463

**Published:** 2010-12-28

**Authors:** Patricia L. Semple, Marcia Watkins, Virginia Davids, Alan M. Krensky, Willem A. Hanekom, Gilla Kaplan, Stanley Ress

**Affiliations:** ^1^Division of Clinical Immunology, Department of Medicine, University of Cape Town, Cape Town 7925, South Africa; ^2^National Health Laboratory Service, Cape Town 8000, South Africa; ^3^South African Tuberculosis Vaccine Initiative, Institute of Infectious Diseases and Molecular Medicine, University of Cape Town, Cape Town 7700, South Africa; ^4^National Cancer Institute, National Institutes of Health, Bethesda, MD 20892-8322, USA; ^5^School of Child and Adolescent Medicine, University of Cape Town, Cape Town, South Africa; ^6^Laboratory of Mycobacterial Immunity and Pathogenesis, Public Health Research Institute, University of Medicine and Dentistry of New Jersey, Newark, NJ 07103-3535, USA; ^7^Groote Schuur Hospital, Cape Town 7925, South Africa

## Abstract

*Background*. While vaccination at birth with *Mycobacterium bovis* Bacilli Calmette-Guérin (BCG) protects against severe childhood tuberculosis, there is no consensus as to which components of the BCG-induced immune response mediate this protection. However, granulysin and perforin, found in the granules of cytotoxic T lymphocytes and Natural Killer (NK) cells, can kill intracellular mycobacteria and are implicated in protection against *Mycobacterium tuberculosis*. *Methods*. We compared the cellular expression of granulysin and perforin cytolytic molecules in cord blood and peripheral blood from 10-week-old infants vaccinated at birth with either Japanese or Danish BCG, administered either intradermally or percutaneously. *Results*. In cord blood, only CD56^+^ NK cells expressed granulysin and perforin constitutively. These cytolytic mediators were upregulated in CD4^+^ and CD8^+^ cord blood cells by *ex vivo* stimulation with BCG but not with PPD. Following BCG vaccination of neonates, both BCG and PPD induced increased expression of granulysin and perforin by CD4^+^ and CD8^+^ T cells. There was no difference in expression of cytolytic molecules according to vaccination route or strain. *Conclusions*. Constitutive expression of perforin and granulysin by cord blood NK-cells likely provides innate immunity, while BCG vaccination-induced expression of these cytolytic mediators may contribute towards protection of the neonate against tuberculosis.

## 1. Introduction

It is estimated that approximately one third of the world's population is infected with *Mycobacterium tuberculosis* (M.tb) resulting in about 1.7 million deaths from tuberculosis (TB) annually [1]. *Mycobacterium bovis* Bacille Calmette Guérin (BCG) is the most commonly used vaccine worldwide and the only vaccine available for the prevention of TB. The vaccine offers good protection against childhood miliary disease and TB meningitis [2, 3] and has been associated with a reduced risk of acute respiratory tract infections in neonates [4]. However, BCG affords very little protection against pulmonary TB and other manifestations of adult disease. It has been suggested that the strain of BCG and the route of administration may affect the efficacy of the vaccine [5–8] as well as determine the nature of the antigen-specific T-cell responses [9]. 

Both innate and adaptive cellular immune responses are required for an effective host defense against M.tb and individuals with defective cell-mediated immunity (CMI) have a predisposition towards developing TB, or towards more severe disease [10]. T-cell derived interferon gamma (IFN-*γ*) is widely recognized as important in antimicrobial protection. This cytokine is essential for the activation of macrophages, a process required to limit the replication of the bacilli in these cells [11, 12]. However, despite having a full repertoire of helper and cytotoxic T-cells, B-cells, and dendritic cells (DC) [13], circulating neonatal lymphocytes are functionally distinct as compared with adults. Consequently, activation is submaximal [14] and IFN-*γ* production in response to mycobacterial antigens is significantly lower in neonates [15, 16]. However, the value of IFN-*γ* as the best correlate of protection against TB has been challenged [17, 18]. Lymphoid cell-derived cytotoxic molecules found in cytotoxic lymphocytes (CTL) and natural killer (NK) cells have also been implicated in contributing to protective immunity against intracellular pathogens. These cells contain granules rich in granulysin, perforin and granzyme, molecules that contribute to lysis of infected cells. Granulysin, a member of the saponin-like family of lipid binding proteins, has also been shown to directly kill extracellular bacilli and, together with perforin, was able to substantially reduce the viability of intracellular M.tb [19].

The aim of our study was to evaluate the expression of granulysin and perforin in NK cells and T-cells in cord blood mononuclear cells (CBMCs) and to compare this with peripheral blood mononuclear cells (PBMCs) from neonates after vaccination with BCG. PBMCs from healthy adults who were purified protein derivative of tuberculin (PPD) responsive *in vitro* were used as controls. In addition, we determined whether the route and strain of BCG vaccination affected the expression of these cytolytic mediators. The results show that while T-cells from CBMCs do not express granulysin or perforin constitutively, BCG vaccination at birth results in strong upregulation of these mediators in the T-cells of 10-week-old infants. NK cells from CBMCs and PBMC of vaccinated infants constitutively expressed both markers. Variation in route and strain of BCG vaccine did not have a significant effect on cytotoxic mediator expression in T-cells or NK cells of PBMC obtained from 10-week-old neonates.

## 2. Material and Methods

### 2.1. Human Subjects and Vaccination

Healthy HIV-negative pregnant women scheduled to undergo elective Ceasarian section, due to previous Ceasarian sections, were recruited for umbilical cord blood (UCB) collection. This population was selected to avoid the effects of labour on immune responses. 

Healthy 10-week-old infants were enrolled at primary care clinics in the Cape Town region. At birth, the babies had received either Japanese BCG (strain 172: Japan BCG Laboratory) or Danish BCG (strain 1331: Statens Serum Institute). The Japanese vaccine was administered either intradermally (JID) or percutaneously (JPC), while the Danish vaccine was given intradermally (DID). Infants with any acute or chronic disease, born to an HIV+ mothers, or living with a person with active tuberculosis, were excluded. A positive ELISA test for HIV, performed on all infants, also resulted in exclusion.

A positive proliferative response to PPD stimulation in peripheral blood (as defined by both microscopic cell clustering and flow cytometric evaluation) was used to identify healthy adults with prior exposure to mycobacterial antigens, and potentially latently infected with M.tb. All volunteers were HIV negative and were clinically well. Human participation was according to the US Department of Health and Human Services and Good Clinical Practice guidelines. This included the protocol approval by the University of Cape Town Research Ethics Committee and by the UMDNJ Institutional Review Board and informed written consent from a parent of the neonate or from the adult volunteers.

### 2.2. Cord Blood Mononuclear Cells (CBMCs)

After inserting a needle with collection tubing into the umbilical vein of the placenta, the blood was allowed to flow by gravity into a standard blood donation bag (Sabax, Johannesburg, South Africa) containing 2000 units of sodium heparin (Sigma- Aldrich, Steinham, Germany). In the laboratory, the UCB was diluted with equal volumes of Ca^+^ and Mg^+^ free phosphate buffered saline (PBS, Bio Whittaker, Walkersville, MD, USA), and mononuclear cells were isolated by density sedimentation using Ficoll-Hypaque (Sigma-Aldrich, Steinham, Germany). Due to the large number of erythroid precursor cells in UCB, the procedure was repeated [20]. After the final wash the CBMCs were suspended in RPMI (Bio Whittkaker) supplemented with 10% human AB serum (Western Province Blood Transfusion Service, South Africa).

### 2.3. BCG-Vaccinated Infant and PPD-Reactive Adult Peripheral Blood Mononuclear Cells (PBMCs)

PBMC were separated as described for CBMCs, except that a single density gradient centrifugation was performed. PBMCs were cryopreserved until analysis: preliminary experiments showed no significant difference between cytolytic mediator expression in freshly isolated PBMCs and cryopreserved PBMCs from the same donors in both adults and 10-week-old infants (data not shown). Cell viability was determined by trypan blue exclusion dye (Sigma Aldrich).

### 2.4. Stimulation of CBMCs and PBMCs

1 × 10^6^ cells/ml were stimulated with 6 *μ*g/ml of PPD (Statens Serum Institute, DM) or 100 IU interleukin-2 (Aldesleukin, Chiron) for 7 days at 37°C in a 5% CO_2_ humidified incubator. Unstimulated cells served as a background control. The duration of lymphoid cell stimulation was validated in preliminary experiments where granulysin and perforin expression was determined ex vivo and 1, 4, 6, 7, and 8/days after PPD and BCG stimulation. Although granulysin and perforin were detected in NK cells ex vivo, little or no expression was seen in CD4^+^ and CD8^+^ T-cells until 4 days after stimulation and optimal expression peaked at day 7 (data not shown). CBMCs and PBMC isolated from adults who were PPD-reactive were also stimulated with BCG (Danish BCG strain 1331, Statens Serum Institute) at a multiplicity of infection (MOI) of 1 : 1 and 5 : 1, respectively, shown to be optimal in preliminary experiments that evaluated cell death with the viability stain 7AAD. The 7-day duration of lymphoid cell stimulation was validated in preliminary experiments (data not shown).

### 2.5. Flow Cytometric Analysis

**Figure 1:**
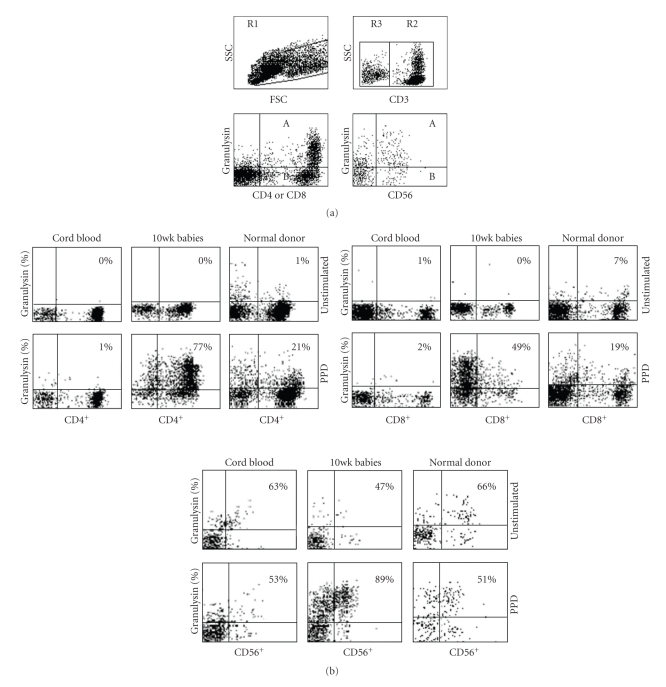
(a) Flow cytometric diagram illustrating the gating strategy for the expression of granulysin (or Perforin) in T-cells and NK cells. R1 represents a general lymphocyte gate including all lymphocytes in a PPD stimulated PBMC sample. R2 incorporates all CD3^+^ T-cells within the R1 gate, and R3 includes all cells that are CD3^−^. Percentage cells expressing granulysin (represented on the y-axis) are evaluated by dividing the number of cells in A by the number of cells in A + B × 100. (b) Flow cytometric dotplots of granulysin in unstimulated or PPD-stimulated representative samples of cord blood mononuclear cells in the left column, 10-week-old infants after BCG vaccination in the middle column, and PBMC from healthy PPD^+^ adult volunteers in the right column. Results shown represent T-lymphocytes which are CD4^+^/CD3^+^ or CD8^+^/CD3^+^ or NK cells which are CD56^+^/CD3^−^. See Section 2 for details.

After stimulation with appropriate stimuli, the cells were washed with PBS containing 1% human AB serum and 0.1% sodium azide (Sigma Aldrich), resuspended in the same medium (wash buffer) and counted on a haemacytometer. Cell viability was determined by trypan blue exclusion (Sigma Aldrich). Approximately 0.1 × 10^6^ viable cells were stained with CD3 FITC (Caltag, Burlingame, CA) or CD3 PerCP (BD Biosciences) and either CD4 APC or CD8 APC (Caltag) or CD56 APC (BD Biosciences) for 15 minutes at room temperature. After washing, the cells were fixed and permeabilised with FACS Permeabilising Solution (BD Biosciences) then stained with either rabbit polyclonal granulysin antibody (which specifically recognises the 9 and 15-kDa forms of granulysin) [21–23] at a concentration of 1/1000 or Perforin-FITC (BD Biosciences) for 30 minutes at 4°C. For granulysin staining, F(ab)_2_ goat antirabbit IgG (H+L)-PE conjugated secondary antibody (Caltag) was added for 30 minutes at 4°C. The cells were resuspended in the wash buffer for analysis on a FACSCalibur using *Cell Quest *software. Isotypic matched controls were used for all monoclonal antibodies while normal rabbit serum (Sigma Aldrich) served as a control for the granulysin polyclonal antibody. The flow cytometric gating strategy (shown in Figure 1(a)) was employed in order to define three distinct peripheral blood lymphocyte subsets for quantification of either granulysin or perforin expression. The lymphocyte gate was defined by forward and side scatter cell characteristics (R1). Within this population, CD3^+^ T-cells were split into CD3^+^4^+^ or CD3^+^8^+^ subsets (i.e., cells that are in both gates R1 and R2), while NK cells were operationally defined as CD3^−^56^+^ (cells that are in both gates R1 and R3) (Figure 1).

### 2.6. Statistical Analysis

The primary outcomes were expression of granulysin or perforin in CD4^+^ or CD8^+^ T-cells, or NK cells. These outcomes were compared according to mycobacterial infection status (naïve cord blood, BCG-vaccinated infants, and healthy PPD-reactive adults) and according to antigen used in assays. The Mann-Whitney two-tailed test or Kruskal Wallis test was used to determine significance of differences, and results are stated throughout the text as medians with interquartile range. All statistical analysis was carried out using either *GraphPad Instat* (version 3.06) or *GraphPad Prism* software (version 4.0).

## 3. Results

### 3.1. Participants

Ten pregnant volunteers with a mean age of 26 years (range 19–34) consented to donating cord blood. Ten PPD-reactive healthy adults (PPD^+^) with a mean age of 36 years (range 24–55) were enrolled in the study. The volunteers comprised of 5 males and 5 females. Forty five babies, vaccinated at birth with BCG, were enrolled in this study: 15 babies received DID whilst 31 babies were vaccinated with Japanese BCG: 15 with JID and 16 with JPC.

### 3.2. Effect of BCG Vaccination on PPD-Induced Granulysin Expression

**Figure 2:**
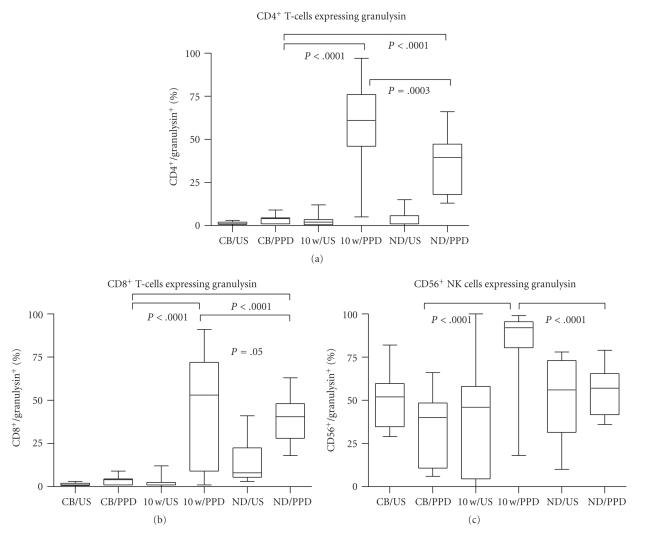
Granulysin expression in CD4^+^ (a) and CD8^+^ (b) T-cells and CD56^+^ (c) NK cells in unstimulated (US) and PPD-stimulated mononuclear cells isolated from cord blood (CB, *n* = 10) or peripheral blood from BCG-vaccinated infants (10 w, *n* = 45) or healthy PPD^+^ adult volunteers (ND, *n* = 10). Significance is shown as PPD stimulation in CB compared to 10-week-old infants and healthy adult controls, and 10-week-old infants compared to healthy adult controls. The box extends from the 25th to 75th percentile, the line represents the median, and the whiskers represent the maximum and minimum values.

CBMCs isolated from placental umbilical veins of 10 neonates were stimulated with PPD for 7 days. CD4^+^ and CD8^+^ T-cells and CD56^+^ NK cells were analysed by flow cytometry for granulysin expression. IL-2 stimulated CBMCs acted as a positive control. Unstimulated and PPD stimulated cord blood CD4^+^ T-cells were either negative or expressed very low levels of granulysin (Figure 2(a)). Similar results were seen in cord blood CD8^+^ T-cells (Figure 2(b)). IL-2 stimulation of CBMCs *in vitro* induced an increase in the percentage of granulysin positive CD4^+^ T cells of 23.5% (12.5–62, *P* =  .0001) and CD8^+^ T cells of 32.5% (10.5–55.5, *P* =  .0001). In contrast to T-cells, unstimulated NK cells expressed high levels of granulysin and the percentage of positive cells was decreased following PPD stimulation although this was not statistically significant (*P* =  .09, Figure 2(c)). Similarly, the level of granulysin in IL-2 stimulated CD56^+^ cells was lower at 20.5% (11.8–32.5), compared to the unstimulated cells at 52% (34.7–59.8), and this was statistically significant (*P* =  .0025, not shown).

When infants were vaccinated with BCG at birth and their PBMC evaluated for granulysin expression at 10 weeks postvaccination, a different pattern was seen. Granulysin was expressed in 2% (0.5–3.5) of unstimulated CD4^+^ and 1% (1–2.5) of unstimulated CD8^+^ T-cells (Figures 2(a) and 2(b)). After 7 days of *in vitro* PPD stimulation of PBMC, granulysin expression in both CD4^+^ and CD8^+^ T-cells increased significantly to a median of 61% (46–76, *P* ≤ .0001) and 53% (9–72, *P* ≤ .0001) of cells, respectively. IL-2 stimulation of PBMC induced similar strong expression with median of 34% (16.5–81, *P* ≤ .0001) for CD4^+^ T cells and 69% (27.5–87, *P* ≤ .0001) for CD8^+^T cell (not shown). Thus, in contrast to CBMCs, PBMC of vaccinated babies had significantly higher levels of granulysin expressing T lymphocytes after *in vitro* PPD stimulation. This high level of expression is comparable to that previously obtained in adults [23]. Similar to CBMCs, constitutive expression of granulysin was seen in unstimulated NK cells of PBMC from vaccinated infants.

Healthy PPD^+^ adult volunteer controls were also evaluated for granulysin expression in NK^+^ cells, and CD4^+^ and CD8^+^ T cells. Unlike both the CBMCs and PBMC from the 10-week-old infants, constitutive granulysin expression was seen in 8% (5.5–22.5) of CD8^+^ T-cells (Figure 2). The median granulysin expression in PPD-stimulated adult PBMC was 39.5% (18–47.3) and 40.5% (28–48) for CD4^+^ and CD8^+^ T cells, respectively, and 57% (41.8–65.5) for NK cells. Compared to unstimulated cells, granulysin expression was significantly increased in PPD-stimulated CD4^+^ (*P* <  .0001) and CD8^+^ (*P* = .0015) T-cells but there was no significant difference in granulysin expression between unstimulated and PPD-stimulated NK cells (*P* = .8). However, similar to CBMCs and PBMC from vaccinated babies, constitutive granulysin expression was also seen in 54.5% (34.8–73) of unstimulated CD56^+^ cells from healthy PPD+ adult controls.

### 3.3. Effect of BCG Vaccination on PPD-Induced Perforin Expression

**Figure 3:**
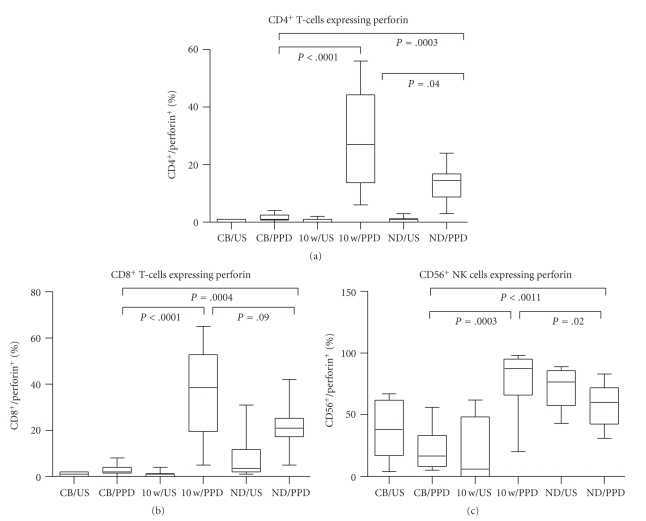
Perforin expression in CD4^+^ (a) and CD8^+^ (b) T-cells and CD56^+^ (c) NK cells in unstimulated (US) and PPD-stimulated mononuclear cells isolated from cord blood (CB, *n* = 10) or peripheral blood from BCG-vaccinated infants (10 w, *n* = 45) or healthy PPD^+^ adult volunteers (ND, *n* = 10). Significance is shown as PPD stimulation in CB compared to 10-week-old infants and healthy adult controls, and 10-week-old compared to healthy adult controls. The box extends from the 25th to 75th percentile, the line represents the median, and the whiskers represent the maximum and minimum values.

A similar analysis was undertaken to determine levels of expression of perforin. Neither unstimulated CD4^+^ nor CD8^+^ T-cells from cord blood, nor unstimulated CD4^+^ or CD8^+^ T cells from 10-week-old vaccinated infants expressed perforin (Figures 3(a) and 3(b)). In contrast, 38% (17–61.8) of NK-cells in CBMCs and 6% (0–48.3) of NK cells in 10-week-old infant PBMC constitutively expressed the cytolytic marker (Figure 3(c)). Again, PPD stimulation did not induce expression of perforin in CD4^+^ or CD8^+^ T-cells from cord blood (Figures 3(a) and 3(b)) while a reduction in the levels of expression in CD56^+^ NK cells to 16.5% (8–33.2, *P* = .02) as compared to unstimulated CBMCs was noted. In contrast, PPD stimulation induced significantly increased expression of perforin in PBMC of vaccinated infants both in CD4^+^ T-cells (*P* = .04) and CD8^+^ T-cells (*P* = .009) (27% (13.8–44.3) and 38.5% (19.5–52.8), resp.) as well as in NK cells (*P* < .0001) (87.5% (66–95)). Compared to CBMCs, there was a significant difference in perforin expression after PPD stimulation in the 10-week-old vaccinated infants in CD4 and CD8 T-cells (*P*, 0.0001 for both T-cells, Figures 2(a) and 2(b)) and in NK cells (*P* = .0003, Figure 3(c)). After IL-2 stimulation, 18% (14–28.5, *P* = .0015) of CD4^+^ T-cells and 36% (22–40, *P* = .002) of CD8^+^ T-cells expressed perforin (not shown).

In PPD^+^ adults, constitutive expression of perforin was seen in 3.5% (2–11.8) of CD8^+^ T-cells and 76.5% (57.5–85.8) of NK cells; after PPD stimulation, 14.5% (8.8–16.8) of CD4^+^ T-cells and 21% (17.3–25.3) of CD8^+^ T-cells expressed perforin. PPD stimulation of NK cells resulted in a reduction of perforin expression (from 76.5% to 60%).

### 3.4. Effect of BCG Stimulation on Granulysin and Perforin Expression in CD4^+^ and CD8^+^ T Cells of CBMCs and in PBMC of PPD^+^ Healthy Adults

**Figure 4:**
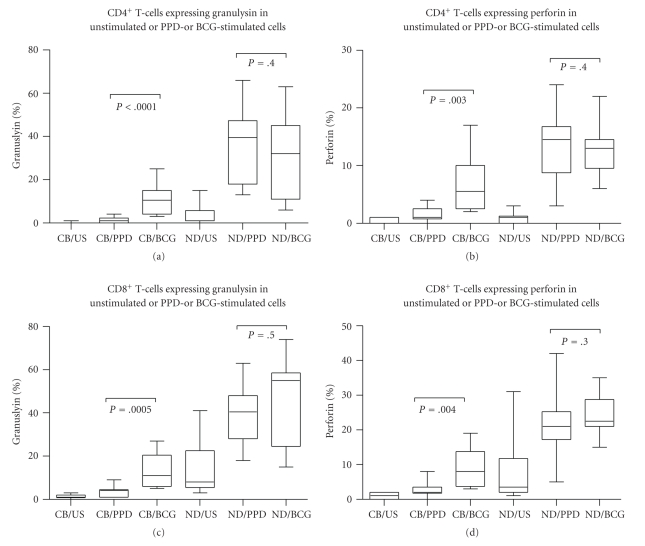
Percentage CD4^+^3^+^, CD8^+^3^+^ expressing Granulysin (a, c), or Perforin (b, d) in unstimulated (US) or PPD- or BCG-stimulated cord blood mononuclear cells (CB *n* = 10) or peripheral blood mononuclear cells from healthy PPD^+^ adult volunteers (ND *n* = 10). Significance is shown between PPD and BCG stimulation in CB and adult volunteers. The box extends from the 25th to 75th percentile, the line represents the median, and the whiskers represent the maximum and minimum values.

PBMC from healthy PPD^+^ adults and CBMCs were stimulated with Danish BCG, and granulysin and perforin expression was determined within the respective CD3^+^ cells. Unlike PPD, BCG stimulation did result in an increase in both granulysin and perforin expressions in CBMCs. There was a statistically significant difference in granulysin expression in BCG-stimulated T-cells compared to PPD-stimulated T-cells (*P* ≤ 0.0001 for CD4^+^ and *P* = .0005 for CD8^+^, Figures 4(a) and 4(c)) in CBMCs. Similarly, perforin-expressing T-cells were also increased in BCG-stimulated compared to PPD-stimulated CBMCs (*P* = .003 for CD4^+^ and *P* = .004 for CD8^+^, Figures 4(b) and 4(d)). 10.5% (4–25) of CD4^+^ T-cells and 11% (6–27) of CD8^+^ T-cells expressed granulysin after BCG stimulation (Figure 4), and perforin expression was seen in 5.5% (2.5–17) and 8% (3.8–19) of CD4^+^ and CD8^+^ T-cells, respectively, after BCG stimulation. Thus, although the percentage of expression was considerably lower than in healthy PPD positive adults, significant expression was induced in both lymphocyte subsets compared to PPD stimulation. In contrast, in PPD*^+^* healthy adult T-cells, equally strong granulysin and perforin expression was obtained with no significant difference between PPD and BCG stimulation being noted (Figures 4(a)–4(d)).

### 3.5. Effect of Route and Strain of the BCG Vaccine on Granulysin and Perforin in T-Cells and NK Cells of PBMCs from 10-Week-Old Infants

To evaluate whether the strain of BCG or the route of administration of the vaccine affected the extent of cytotoxic molecule expression, PBMC from 10-week-old infants vaccinated with either Danish BCG administered intradermally (*n* = 14 granulysin and *n* = 3 for perforin) or Japanese BCG administered either intradermally (*n* = 15 for granulysin and *n* = 4 for perforin) or percutaneously (*n* = 16 granulysin, and *n* = 7 for perforin) were evaluated for granulysin and perforin expression. Although the number of patients evaluated for perforin expression was low, no significant differences were found in granulysin or perforin expression between vaccination with Japanese or Danish BCG. Similarly, the route of administration of the vaccine also did not have any effect on expression of these markers (data not shown).

## 4. Discussion

In the current study, we have evaluated expression of the cytolytic mediators perforin and granulysin in both CD56^+^ NK cells and in CD4^+^ and CD8^+^ T-cell subsets in CBMCs and observed that (i) intrinsic expression of cytolytic mediators was limited to CD56^+^ cells and not found in CD4^+^ and CD8^+^ T-cells and (ii) induction of perforin and granulysin expression in cord blood T-cells occurred selectively in response to *in vitro* stimulation with BCG, but not PPD. In contrast, in 10-week-old BCG-vaccinated infants, upregulation of perforin and granulysin expression in CD4^+^ and CD8^+^ T-cell was demonstrable in response to both BCG and PPD stimulation (data not shown). In this study, the response was independent of variation in vaccine strain or route of vaccine delivery (data not shown), although the sample size was small and not statistically powered. To our knowledge, a comprehensive comparative evaluation of NK and CD4^+^ and CD8^+^ T subset cytolytic mediator expression in cord blood and in BCG vaccinated infants has not been documented before. Previous studies have reported that cord blood CD4^+^ lymphocytes lack constitutive expression of perforin [24] while most CD8^+^ T-cells of newborns have been shown to contain perforin and granzyme. In contrast, we observed that unstimulated cord blood CD8^+^ T-cells did not express granulysin or perforin. This difference may be explained by the fact that in our study the CBMCs were from mothers undergoing caesarean section. During normal vaginal delivery, cytokines including IL-15 may be induced which could activate CD8^+^ T cells to acquire cytotoxic potential [25]. 

Considerable evidence supports the observation that cytotoxic T-cell activation occurs during induction of the host protective immune response to TB [26–28]. We have previously documented CD4^+^ cytotoxic activity in BCG-vaccinated neonates using a chromium release assay [29]. It has previously been shown that the level of expression of cytotoxic mediators is directly related to functional cytotoxic activity of memory CD8^+^ T-cells [30]. Using this approach, we have reported on CD8^+^ cytotoxic molecule expression in infants who had received BCG vaccination at birth [31]. Here we demonstrate that cytotoxic cells expressing granulysin and perforin constitutively exist in neonatal blood even before BCG vaccination. Perforin and granulysin are important cytolytic effector molecules involved in lysis of infected macrophages. Granulysin, found in the granules of cytotoxic T-lymphocytes and NK cells, has a broad spectrum of antimicrobial activity [19]. The molecule has been shown to kill extracellular *M. tuberculosis* and, together with perforin, is bactericidal to intracellular organisms [19]. In addition, granulysin and perforin were significantly increased in lymphoid cells of cattle following vaccination with *M. bovis* BCG and *M. bovis* ΔRD1 compared with nonvaccinated animals [32]. 

IFN-*γ* production by CBMC NK cells is most needed after birth [33, 34], at a time when CD4^+^ CD45RO- T-cells have down-regulated Th1 function by transcriptional regulation of the IFN-*γ* promoter gene due to hypermethylation [35]. Intrinsic cytolytic mediator expression provides a second mechanism for NK cell mediated protective immunity during this critical period of heightened susceptibility to mycobacterial infection. Our observation that BCG upregulates cytotoxic molecules in cord blood CD4^+^ and CD8^+^ T-cells may also be secondary to activation of cells of the innate immune system. Since IL-2 treatment of the T-cells of cord blood directly upregulated perforin and granulysin expression, the effect of BCG could be via activation of NK cells to produce IL-2 which in turn could affect CD4^+^ and CD8^+^ T-cell activation. Support for a central role of NK cells in the host response against TB infection also comes from the bovine model where activated NK cells were shown to have increased granulysin and perforin expression and to lyse *Mycobacterium bovis* BCG-infected alveolar and monocyte-derived macrophages [36]. 

The role of IFN-*γ* producing CD4^+^ T-cells in protection against TB is well described [37–39]. Support for a role for cytotoxic CD4^+^ effectors in protective immunity against TB comes from studies carried out in BCG-vaccinated cattle. In the bovine model, memory CD4^+^ T-cells expressing elevated levels of perforin and granulysin strongly lysed BCG-infected macrophages [40]. Cytotoxic CD4^+^ T-cells have also been shown to use granulysin to kill Cryptococcus neoformans [41]. Furthermore, cytotoxic granulysin-expressing CD4^+^ T-cells have been isolated from skin lesions of tuberculoid leprosy patients [42]. Recently, Murray et al. showed that BCG vaccination induced specific CD8^+^ T-cells which produced IFN-*γ* and had increased expression of cytotoxic proteins in response to BCG stimulation *in vitro, *providing evidence of a role for CD8^+^ T-cells as well [31]. This is supported by the finding of reduced perforin and granulysin coexpression in CD8^+^ T-cells found at the site of infection in chronic TB [43].

In the present study, we observed that there was no statistical difference in granulysin or perforin expression in cells obtained from neonates vaccinated at birth with either JID or DID and no difference between vaccinations via the two routes (intradermal versus percutaneous) of administration of the Japanese BCG. Differences have been reported in a study using a similar but larger cohort of infants, where JPC induced significantly more BCG-specific IFN-*γ* producing CD4^+^ and CD8^+^ T-cells and greater Th1-specific immunity than JID or DID [9]. In addition, JPC induced greater CD4^+^ and CD8^+^ T-cell proliferation. Taken together, the data suggest that distinct factors are involved in promoting the development of the two pathways, that is, Th1 cytokine and T-cell proliferation and expansion of cytotoxic cells expressing granulysin and perforin. In support of this dichotomy, it has been shown that BCG-infected immature DCs selectively expanded perforin-positive CD8^+^ T-cells with little contribution from cytokines, including IFN-*γ*, TNF-*α*, or IL-12 [44]. In contrast, IL-15 promotes granulysin expression [36, 45] while IL-21 has been shown to enhance lytic activity of cytotoxic T-cells and NK cells [46]. These findings may have implications for immunotherapeutic boosting of cytotoxic CD8^+^ activity in TB and BCG vaccination protocols. In addition to therapeutic strategies aimed at inhibition of IL-4 and TGF*β* [47], molecular engineering of BCG to incorporate IL-15 or IL-21 could result in enhanced cytotoxic activity of both NK cells and CD8^+^ cytotoxic T-cells. 

In conclusion, data presented here demonstrate (i) constitutive expression of granulysin and perforin cytolytic mediators in NK cells which could provide innate protective immunity for newborn infants and (ii) that BCG vaccination at birth can induce a cytolytic effector function in neonates, where CD4^+^ and CD8^+^ T-cells are all essentially naïve. By contrast, PPD antigenic stimulation could only induce cytolytic effector functions in memory CD4^+^ and CD8^+^ T-cells. While no currently available immunologic test can predict vaccine efficacy, BCG readily elicits a type 1 cytokine response [29, 48] and it has been proposed that additional factors may be important for vaccine efficacy. These include IL-4 and the Th1 cytokine balance [49, 50] and induction of cytolytic activity in memory T-cells [40, 51]. Further research is needed to establish if BCG-induced cytolytic mediator expression is sustained in memory CTL. It has been postulated that protection due to BCG vaccination wanes with time [52] which may be due to overattenuation of BCG strains with resultant gene deletions [53] and failure to stimulate a durable immune response.

##  Conflict of Interests

 A. M. Krensky holds patents on granulysin as follows: US6485928 Use of granulysin as an antimicrobial agent, US11438563 Antimicrobial peptides, and US4994369 T cell activation related gene (granulysin). P. L. Semple, M. L. V. Watkins, V. Davids, W. A. Hanekom, G. Kaplan, and S. R. Ress have no conflict of interests.
